# Supplementary material for: Functional assignment of Golgi-associated vesicle tethers to specific membrane recycling pathways
Source: bioRxiv. 2026 May 21:2026.05.20.726668. Preprint. [Version 1] doi: 10.64898/2026.05.20.726668 (PMC13228508; doi:10.64898/2026.05.20.726668)
Supplement: 7 [file NIHPP2026.05.20.726668v1-supplement-7.pdf]

bioRxiv preprint doi: https://doi.org/10.64898/2026.05.20.726668; this version posted May 21, 2026. The copyright holder for this preprint (which was not certified by peer review) is the author/funder, who has granted bioRxiv a license to display the preprint in perpetuity. It is made available under a CC-BY 4.0 International license.

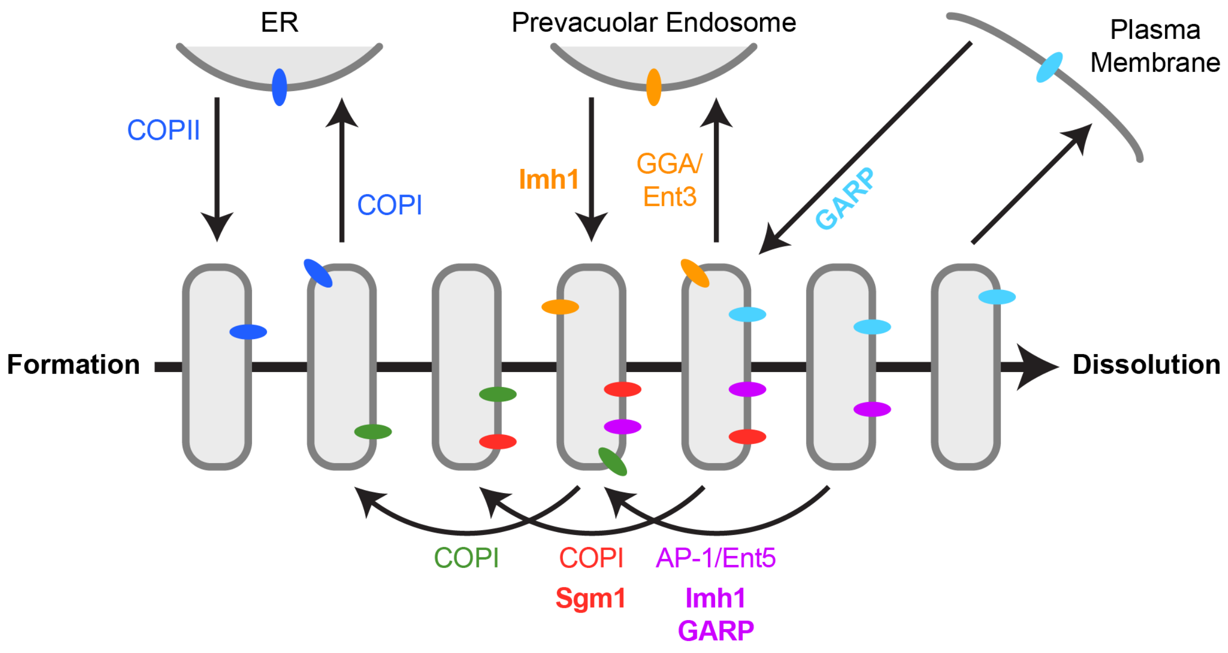

Figure S1. **Multiple recycling pathways deliver membrane to the yeast Golgi with the aid of vesicle tethers.** This diagram summarizes our interpretations about membrane traffic pathways at the yeast Golgi and the involvement of vesicle tethers. The thick arrow represents the timeline of cisternal maturation while the thin arrows represent vesicular traffic pathways. Transmembrane proteins are shown as colored ovals, and the tethers examined here are listed in bold. Six pathways are depicted. (i) COPII vesicles fuse with new Golgi cisternae, and some of the delivered proteins (dark blue) subsequently recycle to the ER in COPI vesicles. (ii) Some resident Golgi proteins such as Vrg4 (green) recycle within the Golgi in a COPI-dependent early pathway. (iii) Some resident Golgi proteins such as Tmn1 (red) recycle within the Golgi in a COPI-dependent intermediate pathway. (iv) Some resident Golgi proteins such as Kex2 (magenta) recycle within the Golgi in an AP-1/Ent5-dependent late pathway. (v) Some proteins such as Vps10 (orange) travel from Golgi cisternae to PVE compartments in a GGA/Ent3-dependent pathway and then recycle to the Golgi. (vi) Some proteins (light blue) travel to the plasma membrane in secretory vesicles and then recycle to the Golgi in endocytic vesicles. Although the endocytic pathway was not characterized in this study, GARP is presumed to tether endocytic vesicles based on evidence that GARP participates in the trafficking of endocytosed proteins (Bonifacino and Hierro, 2011) and that endocytic vesicles in yeast fuse directly with maturing Golgi cisternae (Day et al., 2018).

bioRxiv preprint doi: https://doi.org/10.64898/2026.05.20.726668; this version posted May 21, 2026. The copyright holder for this preprint (which was not certified by peer review) is the author/funder, who has granted bioRxiv a license to display the preprint in perpetuity. It is made available under a CC-BY 4.0 International license.

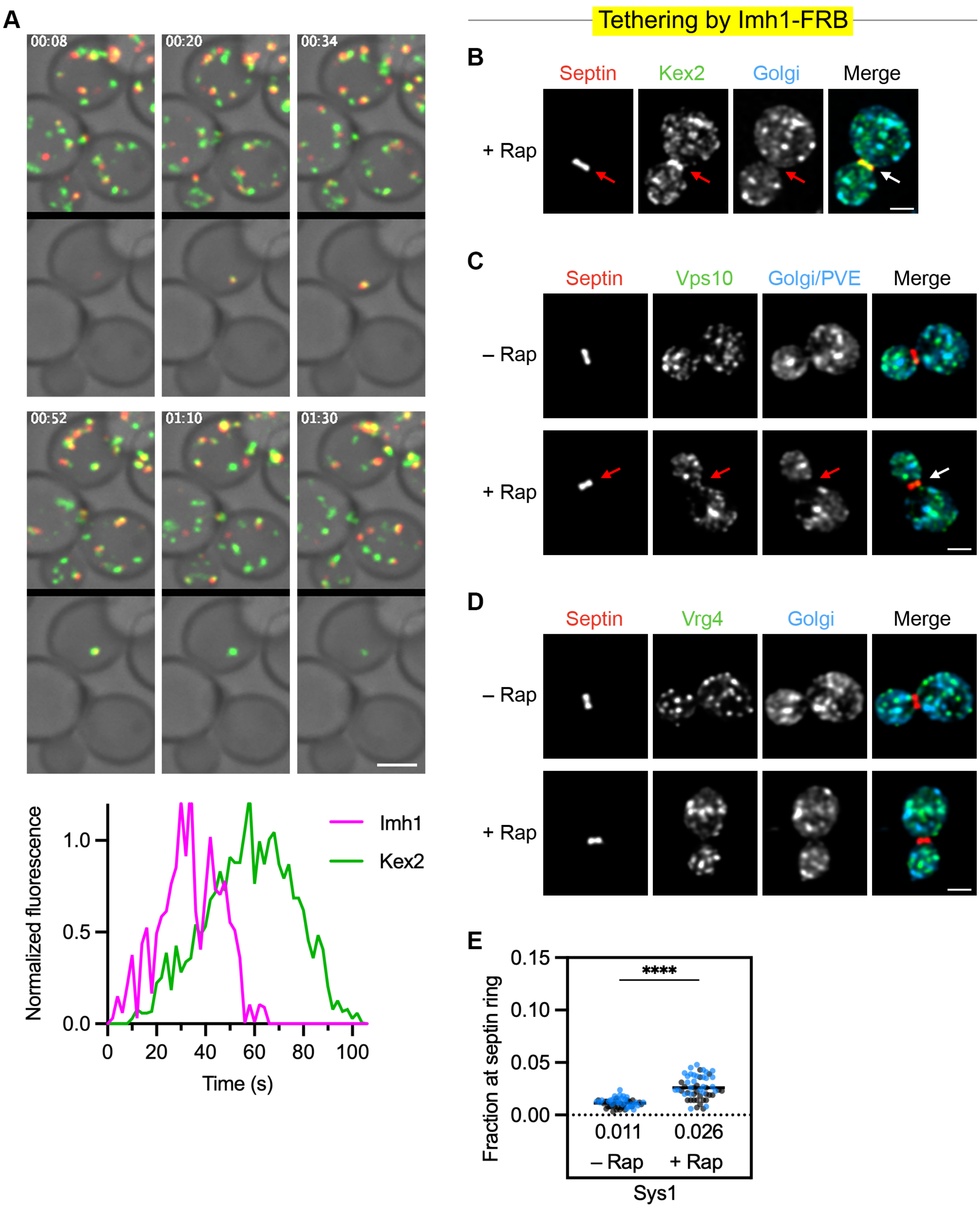

bioRxiv preprint doi: https://doi.org/10.64898/2026.05.20.726668; this version posted May 21, 2026. The copyright holder for this preprint (which was not certified by peer review) is the author/funder, who has granted bioRxiv a license to display the preprint in perpetuity. It is made available under a CC-BY 4.0 International license.

Figure S2. **Imh1 arrives at cisternae shortly before Kex2, and ectopically localized Imh1 tethers Kex2, Vps10, and Sys1. (A)** Frames from a representative 4D confocal movie of HaloTag-Imh1 (red) and Kex2-GFP (green), and kinetic traces from an individual cisterna in the movie. Average projected z-stacks are depicted for the indicated time points from Video 1. The upper row shows the complete projections, and the lower row shows edited projections that include only the cisterna that was tracked. Scale bar, 2 µm. Plotted at the bottom are normalized fluorescence intensities for the cisterna tracked in the movie. **(B)** Example of exceptionally strong tethering of Kex2 by ectopically localized Imh1 after a 10-min incubation with rapamycin ("+ Rap"). The analysis was performed as in Fig. 2 C, and this cell corresponds to the highest data point for the quantification from Fig. 2 C for the replicate shown in black. **(C)** Tethering of Vps10 by ectopically localized Imh1. Representative images show that ectopic localization of Imh1 to the FKBP-tagged septin (red) resulted in rapamycin-dependent accumulation of GFP-tagged Vps10 (green) at the bud neck. HaloTag-labeled Ric1, Sec7, and Vps8 (blue) marked Golgi cisternae and PVE compartments. These images accompany the quantification shown in Fig. 2 D. **(D)** Undetectable tethering of Vrg4 by ectopically localized Imh1. Representative images show that ectopic localization of Imh1 to the FKBP-tagged septin (red) resulted in no rapamycin-dependent accumulation of GFP-tagged Vrg4 (green) at the bud neck. HaloTag-labeled Ric1 and Sec7 (blue) marked Golgi cisternae. These images accompany the quantification shown in Fig. 2 E. **(E)** Tethering of Sys1 by ectopically localized Imh1. Fluorescence at the bud neck for GFP-tagged Sys1 was quantified with or without a 10-min rapamycin treatment as in Fig. 1 B. The total numbers of cells examined were 55 ("– Rap") and 54 ("+ Rap"). ****, significant at P value <0.0001. Multiple regression analysis yielded a P value of 0.0275 that the two replicates were different.

bioRxiv preprint doi: https://doi.org/10.64898/2026.05.20.726668; this version posted May 21, 2026. The copyright holder for this preprint (which was not certified by peer review) is the author/funder, who has granted bioRxiv a license to display the preprint in perpetuity. It is made available under a CC-BY 4.0 International license.

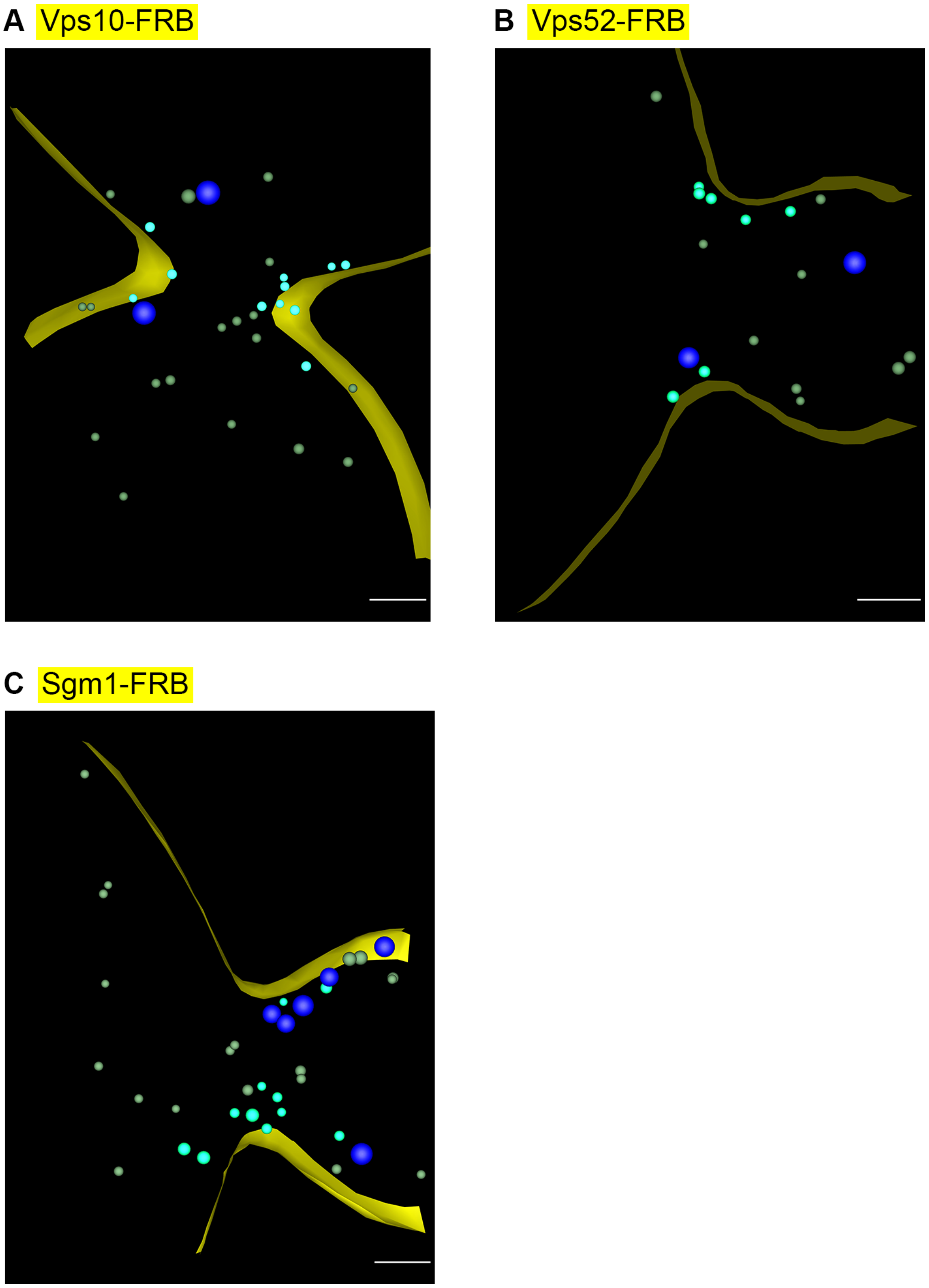

bioRxiv preprint doi: https://doi.org/10.64898/2026.05.20.726668; this version posted May 21, 2026. The copyright holder for this preprint (which was not certified by peer review) is the author/funder, who has granted bioRxiv a license to display the preprint in perpetuity. It is made available under a CC-BY 4.0 International license.

Figure S3. **Cryo-electron tomography confirms that various classes of vesicles can be made to accumulate at the yeast bud neck using the FKBP-rapamycin-FRB system.** Segmented intracellular structures are rendered as in Fig. 1 C. Scale bars, 250 nm. **(A)** Capture of vesicles carrying FRB-tagged Vps10. A log-phase culture of cells expressing Vps10-FRB and Shs1-FKBP was treated with rapamycin for 5 min followed by cryopreservation and processing for cryo-ET. Shown is the model of a SIRT-reconstructed tomogram from a large budded cell. The full data set is shown in Video 3. A vesicle was counted as putatively captured if its membrane was no more than 116 nm from a point on the plasma membrane within 200 nm from the center of the bud neck. For the 4 non-rapamycin-treated cells examined, the number of vesicles meeting this criterion ranged from 0 to 3 (mean = 1.5). For the 4 rapamycin-treated cells examined, the number of putatively captured vesicles ranged from 4 to 12 (mean = 7.0). **(B)** Tethering of vesicles by ectopically localized GARP. A log-phase culture of cells expressing Vps52-FRB and Shs1-FKBP was treated with rapamycin for 10 min followed by cryopreservation and processing for cryo-ET. Shown is the model of a SIRT-reconstructed tomogram from a large budded cell. The full data set is shown in Video 4. A vesicle was counted as putatively tethered if its membrane was no more than 87 nm from a point on the plasma membrane within 200 nm from the center of the bud neck. For the 4 non-rapamycin-treated cells examined, the number of vesicles meeting this criterion ranged from 0 to 2 (mean = 0.8). For the 4 rapamycin-treated cells examined, the number of putatively tethered vesicles ranged from 2 to 7 (mean = 4.3). **(C)** Tethering of vesicles by ectopically localized Sgm1. A log-phase culture of cells expressing Sgm1-FRB and Shs1-FKBP was treated with rapamycin for 10 min followed by cryopreservation and processing for cryo-ET. Shown is the model of a SIRT-reconstructed tomogram from a large budded cell. The full data set is shown in Video 5. A vesicle was counted as putatively tethered if its membrane was no more than 171 nm from a point on the plasma membrane within 200 nm from the center of the bud neck. For the 4 non-rapamycin-treated cells examined, the number of vesicles meeting this criterion ranged from 2 to 5 (mean = 3.3). For the 9 rapamycin-treated cells examined, the number of putatively tethered vesicles ranged from 4 to 13 (mean = 7.7).

bioRxiv preprint doi: https://doi.org/10.64898/2026.05.20.726668; this version posted May 21, 2026. The copyright holder for this preprint (which was not certified by peer review) is the author/funder, who has granted bioRxiv a license to display the preprint in perpetuity. It is made available under a CC-BY 4.0 International license.

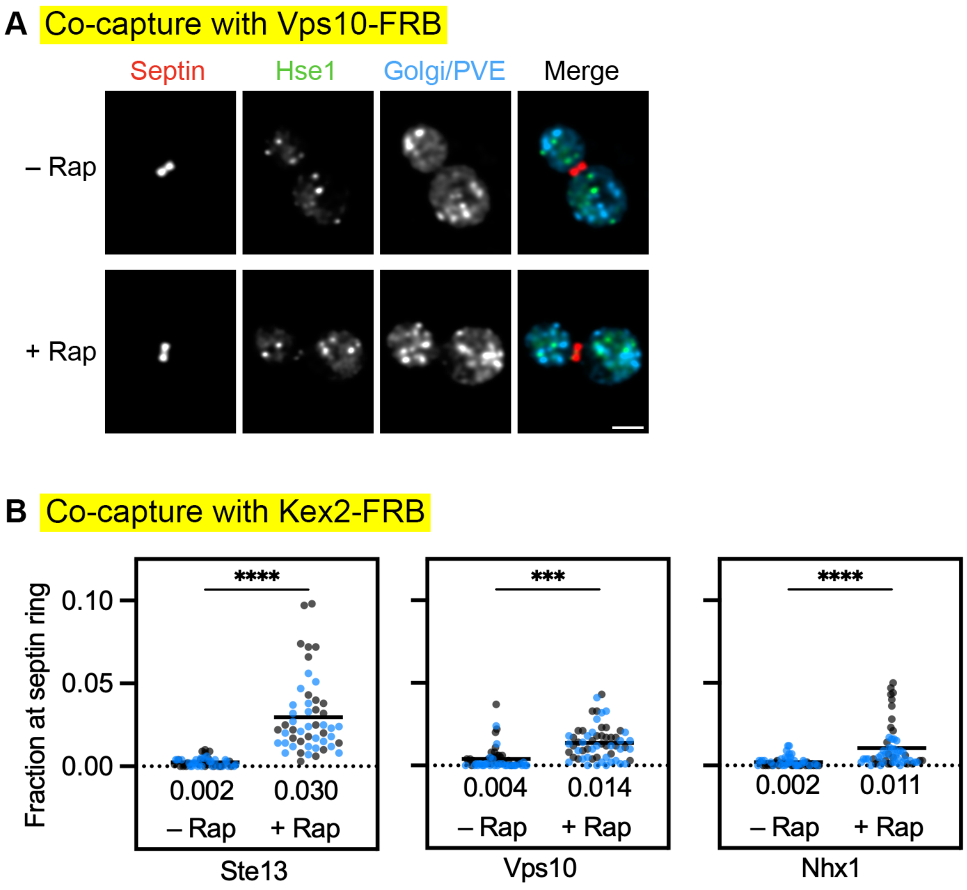

Figure S4. **Vesicle co-capture assays can be used to examine the traffic pathways of TGN proteins. (A)** Control experiment confirming minimal co-capture of the PVE marker Hse1 when Vps10-FRB is captured at the bud neck. Representative images show that capture of Vps10-FRB-containing vesicles at the FKBP-tagged septin (red) resulted in very little rapamycin-dependent accumulation of GFP-tagged Hse1 (green) at the bud neck. HaloTag-labeled Ric1, Sec7, and Vps8 (blue) marked Golgi cisternae and PVE compartments. These images accompany the quantification shown in Fig. 4 B. **(B)** Weak co-capture of GFP-tagged Vps10 or Nhx1 when Kex2-FRB is captured at the bud neck. As a control, GFP-tagged Ste13 shows relatively strong co-capture. Fluorescence at the bud neck was quantified with or without a 5-min rapamycin treatment as in Fig. 1 B. The total numbers of cells examined were 51 (Ste13 "– Rap"), 50 (Ste13 "+ Rap"), 55 (Vps10 "– Rap"), 60 (Vps10 "+ Rap"), 56 (Nhx1 "– Rap"), and 55 (Nhx1 "+ Rap"). Multiple regression analysis yielded a P value of 0.0002 that the two Nhx1 replicates were different. ****, significant at P value <0.0001; ***, significant at P value = 0.0002 (Vps10). The Ste13 co-capture data are reproduced from Krahn et al. (2026), originally published in the Journal of Cell Biology (https://doi.org/10.1083/jcb.202509174).

bioRxiv preprint doi: https://doi.org/10.64898/2026.05.20.726668; this version posted May 21, 2026. The copyright holder for this preprint (which was not certified by peer review) is the author/funder, who has granted bioRxiv a license to display the preprint in perpetuity. It is made available under a CC-BY 4.0 International license.

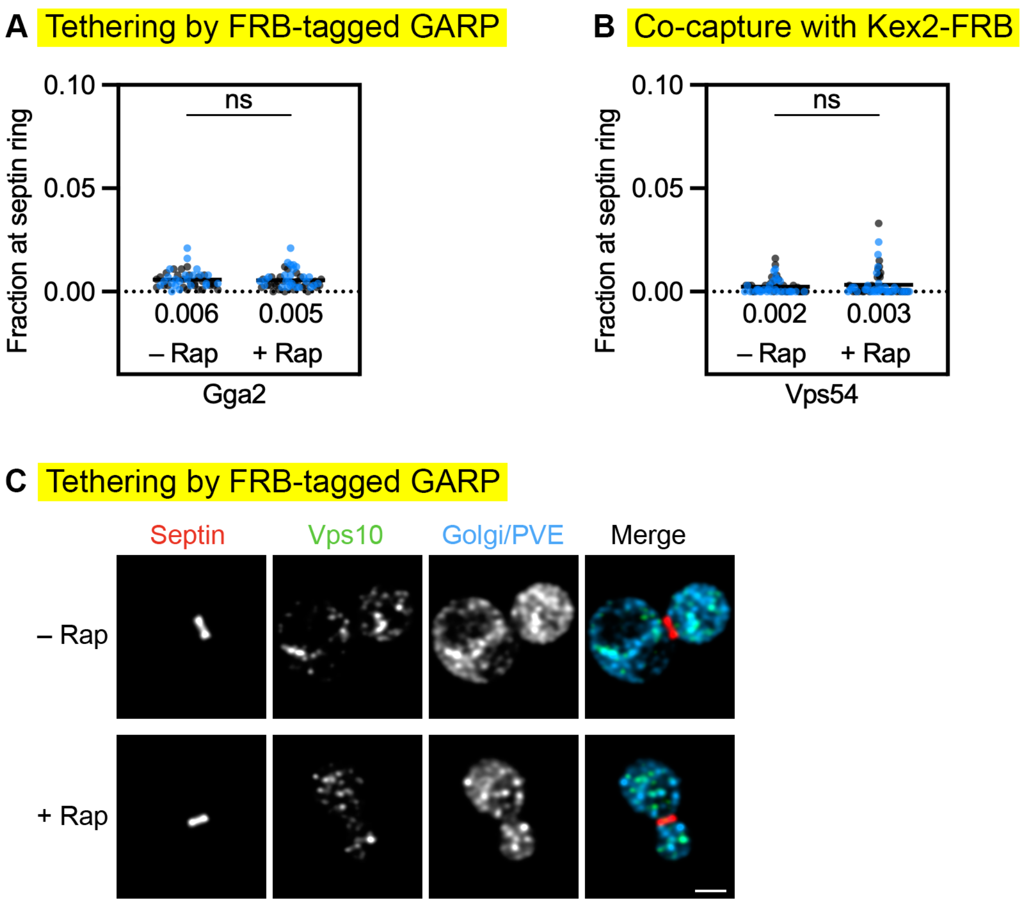

Figure S5. **Control experiments confirm the specificity of tethering by ectopically localized GARP. (A)** Control experiment showing that ectopic localization of GARP (Vps52-FRB) to the FKBP-tagged septin did not result in detectable fluorescence at the bud neck from TGN cisternae marked by Gga2. Fluorescence of GFP-tagged Gga2 at the bud neck was quantified with or without a 10-min treatment with rapamycin ("Rap") as in Fig. 1 B. The total numbers of cells examined were 49 ("– Rap") and 54 ("+ Rap"). ns, not significant. **(B)** Control experiment showing that capture of vesicles containing Kex2-FRB did not result in co-capture of the GARP subunit Vps54. Fluorescence of GFP-tagged Vps54 at the bud neck was quantified with or without a 5-min rapamycin treatment as in Fig. 1 B. The total numbers of cells examined were 58 ("– Rap") and 60 ("+ Rap"). ns, not significant. **(C)** Undetectable tethering of Vps10 by ectopically localized GARP. Representative images show that ectopic localization of GARP (Vps52-FRB) to the FKBP-tagged septin (red) resulted in no rapamycin-dependent accumulation of GFP-tagged Vps10 (green) at the bud neck. HaloTag-labeled Ric1, Sec7, and Vps8 (blue) marked Golgi cisternae and PVE compartments. These images accompany the quantification shown in Fig. 6 B.

bioRxiv preprint doi: https://doi.org/10.64898/2026.05.20.726668; this version posted May 21, 2026. The copyright holder for this preprint (which was not certified by peer review) is the author/funder, who has granted bioRxiv a license to display the preprint in perpetuity. It is made available under a CC-BY 4.0 International license.

Video 1. **Representative 4D confocal movie of HaloTag-Imh1 and Kex2-GFP.** 3D z-stacks for the individual time points were average projected. The upper row shows the complete projections, and the lower row shows edited projections that include only the cisterna that was tracked. Intervals between frames are 2 s. The overlaid numbers represent the time in seconds after the cisterna that was tracked first became detectable. See Fig. S2 A for further details.

bioRxiv preprint doi: https://doi.org/10.64898/2026.05.20.726668; this version posted May 21, 2026. The copyright holder for this preprint (which was not certified by peer review) is the author/funder, who has granted bioRxiv a license to display the preprint in perpetuity. It is made available under a CC-BY 4.0 International license.

Video 2. **Tomographic sections and modeling of part of the bud neck region in a cell with ectopically localized Imh1-FRB bound to an FKBP-tagged septin.** A log-phase culture of cells expressing Imh1-FRB and Shs1-FKBP was treated for 10 min with rapamycin prior to cryopreservation and processing for cryo-ET. The first third of the video shows every fifth section of the SIRT-reconstructed tomogram. The second third of the video shows the same tomographic sections after contours were segmented to mark the cell cortex (yellow), five secretory vesicles (blue), putatively tethered non-secretory vesicles (bright green), and other non-secretory vesicles (dull green). Also marked are a mitochondrion (cyan) and the nuclear envelope (magenta). The final third of the video shows a rotation of the tomographic model. Scale bar, 250 nm.

bioRxiv preprint doi: https://doi.org/10.64898/2026.05.20.726668; this version posted May 21, 2026. The copyright holder for this preprint (which was not certified by peer review) is the author/funder, who has granted bioRxiv a license to display the preprint in perpetuity. It is made available under a CC-BY 4.0 International license.

Video 3. **Tomographic sections and modeling of part of the bud neck region in a cell with Vps10-FRB-containing vesicles captured by an FKBP-tagged septin.** A log-phase culture of cells expressing Vps10-FRB and Shs1-FKBP was treated for 5 min with rapamycin prior to cryopreservation and processing for cryo-ET. The first third of the video shows every fifth section of the SIRT-reconstructed tomogram. The second third of the video shows the same tomographic sections after contours were segmented to mark the cell cortex (yellow), two secretory vesicles (blue), putatively tethered non-secretory vesicles (bright green), and other non-secretory vesicles (dull green). Also marked are a mitochondrion (cyan) and the nuclear envelope (magenta). The final third of the video shows a rotation of the tomographic model. Scale bar, 250 nm.

bioRxiv preprint doi: https://doi.org/10.64898/2026.05.20.726668; this version posted May 21, 2026. The copyright holder for this preprint (which was not certified by peer review) is the author/funder, who has granted bioRxiv a license to display the preprint in perpetuity. It is made available under a CC-BY 4.0 International license.

Video 4. **Tomographic sections and modeling of part of the bud neck region in a cell with ectopically localized Vps52-FRB bound to an FKBP-tagged septin.** A log-phase culture of cells expressing Vps52-FRB and Shs1-FKBP was treated for 10 min with rapamycin prior to cryopreservation and processing for cryo-ET. The first third of the video shows every fifth section of the SIRT-reconstructed tomogram. The second third of the video shows the same tomographic sections after contours were segmented to mark the cell cortex (yellow), two secretory vesicles (blue), putatively tethered non-secretory vesicles (bright green), and other non-secretory vesicles (dull green). Also marked are two mitochondria (cyan) and the nuclear envelope (magenta). The final third of the video shows a rotation of the tomographic model. Scale bar, 250 nm.

bioRxiv preprint doi: https://doi.org/10.64898/2026.05.20.726668; this version posted May 21, 2026. The copyright holder for this preprint (which was not certified by peer review) is the author/funder, who has granted bioRxiv a license to display the preprint in perpetuity. It is made available under a CC-BY 4.0 International license.

Video 5. **Tomographic sections and modeling of part of the bud neck region in a cell with ectopically localized Sgm1-FRB bound to an FKBP-tagged septin.** A log-phase culture of cells expressing Sgm1-FRB and Shs1-FKBP was treated for 10 min with rapamycin prior to cryopreservation and processing for cryo-ET. as treated for 10 min with rapamycin prior to cryopreservation and processing for cryo-ET. The first third of the video shows every fifth section of the SIRT-reconstructed tomogram. The second third of the video shows the same tomographic sections after contours were segmented to mark the cell cortex (yellow), six secretory vesicles (blue), putatively tethered non-secretory vesicles (bright green), and other non-secretory vesicles (dull green). Also marked are a mitochondrion (cyan) and the nuclear envelope (magenta). The final third of the video shows a rotation of the tomographic model. Scale bar, 250 nm.
